# Resting Blood Pressure in Collegiate Swimmers During a Competitive Season: A Prospective Observational Study

**DOI:** 10.7759/cureus.12340

**Published:** 2020-12-28

**Authors:** Sara K Arena, Scott Jones, Anthony M Munoz, Meghan Murley, Ciera (Strudwick) Melton, Kwame Sakyi, Tamara Hew-Butler

**Affiliations:** 1 Physical Therapy, Oakland University, Rochester, USA; 2 Public and Environmental Wellness, Oakland University, Rochester, USA; 3 Exercise and Sport Science, College of Education, Wayne State University, Detroit, USA

**Keywords:** blood pressure, swimmers, athletes

## Abstract

Introduction

The purpose of this study was to describe and examine differences in resting blood pressure (BP) during an eight-week time frame in the course of the competitive season among collegiate swimmers of varied sexes.

Methods

A prospective observational study using a sample of convenience of National Collegiate Athletic Association (NCAA) Division 1 female and male swimmers from one university were invited to participate. Blood pressure was measured using standardized methodology at six encounters spaced over eight weeks. Descriptive statistics analyzed demographics, mean BP, and BP classifications. A pairwise t-test analyzed differences in the mean BP and BP classification by sex. The Bonferroni correction was applied given the multiple variables included in the analysis with statistical significance determined to be p≤0.002.

Results

Thirty-eight swimmers (15 males and 23 females) met the inclusion criteria. Differences between sexes were identified with a higher mean diastolic BP observed in males at the third encounter (p=0.0004) and a higher mean systolic BP observed in males at the sixth encounter (p=0.0002). Four males and four females were identified with a BP classified as stage 1 or 2 hypertension at the first encounter; however, six males and no females met this criterion at the last encounter which was statistically significant (p=0.0004).

Conclusions

Increased BP from baseline measured for systole, diastole, and BP classifications was significant in male compared to female swimmers. Specifically, divergence in BP by sex first appeared in the diastolic measures at three weeks and in the systolic measures and BP classifications by eight weeks.

## Introduction

Sustained elevation in blood pressure (BP) is predictive of future cardiovascular diseases [1]. While increased physical activity may result in lowering or slowing the risk for BP elevation over time [1], prior evidence suggests this measure may actually increase over the course of a competitive season among collegiate athletes [2]. Berge et al. have proposed the high-intensity exercise dosing associated with a conditioning and training regimen of collegiate athletes to be a contributor, however, definitive causation for this occurrence has not been described [3].

While negative short- and long-term health-related outcomes of elevated BP measures have not specifically been examined in the collegiate athlete population, the occurrence of malignant heart arrhythmias, excessive left ventricular hypertrophy, and even sudden cardiac death have been observed [4,5]. These negative health consequences are often not preceded by symptoms and may occur despite no significant findings on pre-season and clinical screening inclusive of electrocardiography and exercise stress testing [4]. Therefore, it is plausible that BP elevation in collegiate athletes’ overtime could be a screening tool to aid in the detection of impending cardiovascular abnormalities.

Stanfa et al. described the occurrence of exertional rhabdomyolysis among National Collegiate Athletic Association (NCAA) swimmers in peak physical condition after a difficult upper body competitive-style training session [6]. Notably, the athletes in this cohort who required hospitalization for rhabdomyolysis related side effects were statistically more likely to have had an increase in resting BP between pre- and post-season BP measures during that same season. Furthermore, water sport athletes have been reported to have higher BP measures than their dry-land athlete counterparts which emphasize the need for further examination of BP in this specific population of athletes [7]. Both male and female collegiate swimmers have been reported to have the highest conversion rates of a normal BP reading (<120 mm Hg systolic/< 80 mm Hg diastolic) to either an elevated (120-129 mm Hg systolic/<80 mm Hg diastolic) or a stage 1 hypertension (HTN) (130-139 mm Hg systolic and/or 80-89 mm Hg diastolic) classification when compared to collegiate athletes from men’s soccer, women’s volleyball and men’s and women’s cross country [8].

Literature suggests BP may increase over a collegiate competitive season among swimmers [2,6]. However, there is a paucity of evidence reporting BP measures at intervals throughout the course of the collegiate swimmer’s season thereby capturing the point at which these BP elevations may be occurring. Additionally, it is unknown if BP variations follow congruent patterns of variations in males and females. A healthcare provider's understanding of BP measures and the associated changes over time may be significant in detecting situations for which these measures may be outside expected values or trends. The purpose of this study was to describe and examine differences in resting BP during an eight-week time frame in the course of the competitive season among collegiate swimmers of varied sexes. This work was previously presented as a research platform presentation at the 2020 American Physical Therapy Associations Combined Section Meeting on February 13, 2020.

## Materials and methods

Study design

Following Oakland University Institutional Review Board (IRB) approval, NCAA Division I athletes from one university in Southeast Michigan were invited to participate in a prospective observational study using a sample of convenience.

Setting

The initial investigation encounter for each collegiate swimmer occurred between late August and early September 2016 in the Prevention Research Center on the Campus of Oakland University in Rochester, MI.

Participants

All members of the 2016-2017 Oakland University men and women swimming and diving (SD) teams were informed of the study by the team coaches or representatives. Swimmers were excluded if they did not attend at least four measurement encounters over a minimum of five weeks. Additionally, athletes were excluded if they reported: (1) performing exercise in the 30 minutes prior to data collection, (2) a current illness, (3) consuming nicotine in the 48 hours prior to the measurement encounter, (4) recent caffeine consumption, and/or (5) being prescribed medications with BP altering effects (i.e., lisinopril, metoprolol) at the time of data collection. 

Procedure

After a physician deemed each athlete healthy during a pre-season physical, investigators secured informed consent to assure the rights of the athlete were protected. During the initial visit, which correlated with the beginning of the competitive season (September through March), the investigators recorded sex and age. Prior to obtaining BP measures, athletes' self-reported responses to the time of most recent workout, current illness, recent tobacco use, recent caffeine intake, and current medication consumption to assure inclusion criteria was met. Additionally, athletes self-reported current or past cardiac disease or arrhythmia.

There were six possible data collection encounters which were initially scheduled to occur once per week on the same day of the week. However, due to competition scheduling differences between male and female athletes encounters five and six were scheduled on different weeks. Specifically, both sexes were offered data collection encounters on August 31, September 9, 16, and 23. However, the final male data collection occurred on September 30 and October 14: whereas, the final female data collection occurred on October 7 and 21. All data were collected in 2016. While athletes continued in their competitive season through March, travel and academics schedules limited data collection beyond October 21, 2016.

The BP readings were obtained by investigators trained in the study protocol and who had demonstrated proficiency with measuring BP using a simulation training arm and a content expert. Auscultatory technique with an appropriately sized and calibrated American Diagnostic Corporation Brand aneroid sphygmomanometer and a Littman Brand Master Classic II (3M Health Care, St. Paul, MN) or Electronic Model 3100 Stethoscope (3M Health Care, St. Paul, MN) was used to obtain all BP measures.

Each encounter, data collectors instructed athletes to sit resting with their back supported, feet uncrossed and flat on the floor, and right arm supported at the level of the heart using an adjustable table to assure the standardized height [8,9]. After maintaining this position at rest for a minimum of five minutes the pulse obliteration pressure was determined followed by two BP measurements at the right brachial artery one minute apart. The average of the two readings for each encounter was used for analysis.

Blood pressure readings on each encounter were classified into one of four categories using the American College of Cardiology (ACC) and American Heart Association (AHA) Task Force recommendations [1]. The systolic and diastolic BP (SBP, DBP) measures were used to determine the classification as follows: normal (SBP less than 120 mm Hg and DBP less than 80 mm Hg), elevated (SBP between 120-129 mm Hg and DBP less than 80 mm Hg), stage 1 HTN (SBP between 130-139 mm Hg or DBP between 80-89 mm Hg) or stage 2 HTN (SBP greater than or equal to 140 mm Hg or DBP greater than or equal to 90 mm Hg) [1].

Statistical analysis

Descriptive statistics reported athlete demographics, mean BP, and BP classifications. A pairwise t-test analyzed differences in the mean BP and BP classification by sex. The authors recognize the use of a sample of convenience may result in an underpowered analysis. However, the potential sample pool was adequate to provide initial observational descriptive reporting and report emerging data trends. Statistical significance was set at p ≤ 0.05; however, due to multiple variables the Bonferroni Correction was applied and a more rigorous significance of p ≤ 0.002 was also examined. Data were analyzed using TIBCO® Statistica (TIBCO Software Inc., Palo Alto, CA).

## Results

Demographics

Thirty-eight swimmers met the inclusion criteria; 15 males and 23 females. It was noted that four males and seven females were unable to attend the first encounter visit for data collection due to academic conflicts; however, given they were participating in team workouts and training sessions data reporting begins in week two for these athletes. Therefore, week one reporting includes data from 27 athletes. The mean age of swimmers was 19.8 (±1.4) and ranged from 17-23 years old. No swimmers reported exercising in the 30 minutes prior to data collection, current illness, recent tobacco use, recent caffeine intake, and/or consumption of prescribed BP altering medication. In addition to physician medical clearance prior to participation, all athletes denied active or a medical history of cardiac disease or abnormal electrocardiography.

Descriptive data

The mean resting SBP and DBP for all swimmers and by sex are reported in Table 1.

**Table 1:** Mean blood pressure of all swimmers and by sex SD=standard deviation

		Week 1	Week 2	Week 3	Week 4	Week 5	Week 6
Systolic blood pressure in mm Hg (SD)	All athletes	120(±15)	119(±10)	118(±9)	119(±11)	117(±8)	117(±10)
	Males	124(±14)	124(±10)	122(±10)	124(±11)	121(±9)	124(±10)
	Females	117(±16)	115(±8)	115(±8)	115± (9)	114(±6)	112(±7)
Diastolic blood pressure in mm Hg (SD)	All athletes	67(±11)	73(±11)	71(±9)	70(±10)	68(±9)	67(±10)
	Males	66(±13)	77(±8)	78(±6)	73(±9)	73(±10)	72(±8)
	Females	67(±10)	70(±12)	67(±9)	67(±10)	65(±9)	63(±9)

The percentage of swimmers categorized in each BP classification for all swimmers and by sex is reported in Table 2.

**Table 2:** Blood pressure classifications of all swimmers and by sex at each encounter HTN=hypertension

Encounter	Blood Pressure Classification	All Athletes % (N)	Males % (N)	Females % (N)
1	Normal	51.9(14)	45.5(5)	56.3(9)
	Elevated	18.5(5)	18.2(2)	18.8(3)
	Stage 1 HTN	14.8(4)	18.2(2)	12.5(2)
	Stage 2 HTN	14.8(4)	18.2(2)	12.5(2)
2	Normal	47.4(18)	26.7(4)	60.9(14)
	Elevated	28.9(11)	40.0(6)	21.7(5)
	Stage 1 HTN	15.8(6)	20.0(3)	13.0(3)
	Stage 2 HTN	7.9(3)	13.3(2)	4.3(1)
3	Normal	50.0(19)	26.7(4)	6.2(15)
	Elevated	28.9(11)	40.0(6)	21.7(5)
	Stage 1 HTN	18.4(7)	26.7(4)	13.0(3)
	Stage 2 HTN	2.6(1)	6.7(1)	0
4	Normal	52.0(13)	30.8(4)	75.0(9)
	Elevated	20.0(5)	30.8(4)	8.3(1)
	Stage 1 HTN	24.0(6)	30.8(4)	16.7(2)
	Stage 2 HTN	4.0(1)	7.7(1)	0
5	Normal	68.4(26)	40.0(6)	87.0(20)
	Elevated	18.4(7)	40.0(6)	4.3(1)
	Stage 1 HTN	7.9(3)	6.7(1)	8.7(2)
	Stage 2 HTN	5.3(2)	13.3(2)	0
6	Normal	60.5(23)	33.3(5)	78.3(18)
	Elevated	23.7(9)	26.7(4)	21.7(5)
	Stage 1 HTN	18.4(7)	33.3(5)	0
	Stage 2 HTN	2.6(1)	6.7(1)	0

Of the 27 swimmers who were assessed during the first encounter visit, 21 had no change in BP classification from the first encounter to the sixth encounter; however, six converted to a more elevated BP classification and 11 converted to a less elevated BP classification. Five males and four females were identified with a BP classified as stage 1 or 2 HTN at the first encounter; however, eight males and no females met this criterion at the final encounter. Lastly, during the last four encounters, all swimmers identified with stage 2 HTN were male. 

Differences by sex

Male swimmers had significantly higher SBP measurements than females at encounters two (p=0.01), three (p=0.02), four (p=0.03), five (p=0.01), and six (p=0.0002); DBP at encounters three (p=0.0004), five (p=0.01), and six (p=0.01); and BP classification at encounters three (p=0.02), five (p=0.01), and six (p=0.0004). It is notable these differences also met the rigor of the Bonferroni Correction at the third encounter for the DBP measures and the sixth encounter for the SBP measures and the BP classification. 

## Discussion

This study aims to describe and examine differences in resting BP over an eight-week time frame during the competitive season among collegiate swimmers of varied sexes. To this end, elevated, stage 1, and stage 2 HTN BP measures in male collegiate swimmers were identified and are findings in congruence with prior evidence among high-level athletes of varied sports and professional dancers [2,10]. Specifically, prior reports suggest elevated BP in male athletes as compared to female may continue into middle age and point toward androgens' role in BP regulation as a potential causative factor [2,11-13]. However, a novel aspect of the current study was that it examined weekly changes in the BP measures of swimmers during in-season training. The identified significantly higher BP measures in male athletes compared to female athletes appeared at approximately three to four weeks after the start of in-season training with the DBP measures being most significant in this time frame. The elevated BP in males compared to females continued to be significant at the final encounter which was approximately seven weeks after the initial encounter and after competitive meets had commenced. During the latter encounters, SBP measure and BP category differences became more pronounced.

While there is limited prior evidence examining the point at which BP variations among sexes occur in athletes, findings of a prior study may offer a potential hypothesis for this occurrence and suggest androgens as potential causation as has been previously discussed. Specifically, Ratamess et al. examined testosterone levels throughout a competitive season (four months) in collegiate wrestlers and identified significantly reduced testosterone levels in the athletes when comparing measurements taken prior to pre-season training versus in-season measurements (13.9% reduction prior to first competition) [14]. Although the present study did not specifically examine the correlation between BP measures and testosterone levels in collegiate-aged athletes it is noteworthy that the changes follow similar time frame patterns. Additionally, an inverse association between testosterone levels and SBP has been previously reported among males [15].

While there is confounding research on the role of estrogens in cardiovascular protection, studies suggest androgens, including testosterone, may contribute to cardiovascular-related diseases including HTN [16]. A study reviewing available evidence of sex and gender differences in BP control indicates reduced androgen levels may indeed exacerbate cardiovascular disease in men [15]. Furthermore, there is evidence of lower BP measures among adolescent females compared to males suggesting these differences may appear in puberty and persist into the sixth decade of life [15,17,18]. This prior evidence supports hormonal variations as a potential rationale for the BP variations observed between the sexes of the collegiate swimmers in our study.

Study limitations

The authors acknowledge limitations to this study including a small sample size from one university and the study design did not utilize a non-athletes control group or control for confounding variables such as stress which could impact resting BP. Additionally, as the data were obtained from water athletes it cannot be generalized to a dry land athlete cohort. Furthermore, weight, height, body mass index, and consumption of medications that may result in hormonal variation inclusive of contraceptive medication were not examined. Finally, the eight-week data collection time frame may limit the ability to realize long term trends in BP variation.

Future research

Future research with a larger sample size would be beneficial to assuring adequate sample sizes over time given the likelihood of athlete attrition. This may necessitate recruitment from multiple collegiate institutions which would have a dual benefit in generalization to a wider scope of training regimens, such as competitive distance or dominant stroke swam. Additionally, a comparison of collegiate water sport athletes to non-athletes and dry land athletes would be beneficial in determining similarities and differences in the BP readings. Furthermore, investigation over a longer time period would be advantageous to examine seasonal variations of BP readings and the potential long-term impact of the elevated BP identified in males. Finally, studies to examine hormonal-BP interaction in collegiate swimmers may be of benefit.

## Conclusions

Increased BP from baseline measures in SBP, DBP, and BP classifications was significant in male compared to female swimmers and are consistent with the prior reports among collegiate athletes of varied sports. Furthermore, BP measure differences between the sexes became more pronounced over time. While future research to determine causative factors for this observation are warranted, the role of testosterone levels in male is hypothesized. However, this is the first study to report a time frame at which the BP measure separation between the sexes of athletes appears. Specifically, divergence in BP by sex first appeared at three weeks and continued throughout the time frame. Findings of this study support the inclusion of BP measures as a component of routine screening and healthcare in collegiate athletes to assure baseline and seasonal variations are evaluated.
